# Relative Contributions of Different Lifestyle Factors to Health-Related Quality of Life in the Elderly

**DOI:** 10.3390/ijerph15020256

**Published:** 2018-02-03

**Authors:** Xiaona Zhang, Ruyi Xia, Shu Wang, Wei Xue, Jian Yang, Shuliu Sun, Guihua Zhuang

**Affiliations:** Department of Epidemiology and Biostatistics, School of Public Heath, Xi’an Jiaotong University Health Science Center, No. 76 West Yanta Road, Xi’an, 710061, China; ppflyman1@stu.xjtu.edu.cn (X.Z.); xiaruyi0820@163.com (R.X.); wangshu_89022table2@126.com (S.W.); 15829632884@163.com (W.X.); yjiann@foxmail.com (J.Y.); shuliu0823@outlook.com (S.S.)

**Keywords:** health-related quality of life, the SF-36v2 Health Survey, the WHOQOL-OLD module, older people, lifestyle, hierarchical regression

## Abstract

Much of the previous literature has studied the relationship between individual lifestyle factors and the health-related quality of life (HRQOL). However, only a few studies combined them to explore their relative importance to the HRQOL in the elderly. This study assesses the HRQOL of the urban, rural, and institutionalized Chinese elderly and explores the relative contributions of different lifestyle factors to their HRQOL. The SF-36v2 Health Survey, the WHOQOL-OLD module, and the socio-demographic and lifestyle questionnaire were utilized in this study. Hierarchical regression was performed in order to analyze the results. The physical and mental component scores of the SF-36v2 survey were 47.05 ± 9.95 and 54.92 ± 9.92, respectively. The total score for the WHOQOL-OLD module was 73.01 ± 11.99, with institutionalized persons reporting lower scores. For the physical component of the elderly participants’ HRQOL, the R^2^ value changed the most (0.116) when exercise-and-labor-related factors were added in. For the mental component, sleep-related (0.054), and leisure-time-activity-related factors (0.053) caused the largest change of the R^2^ value. For the elderly-specific HRQOL, measured by the WHOQOL-OLD module, the leisure-time-activity-related factors caused the largest change in the R^2^ value (0.119), followed by exercise-and-labor-related factors (0.078). Heterogeneity was present among the three subgroups. In sum, compared with their community-dwelling counterparts, the HRQOL of institutionalized older people was relatively poor and different lifestyle factors contributed to the HRQOL differently.

## 1. Introduction

The human population is aging rapidly around the world. According to the World Health Organization (WHO), between 2015 and 2050, the proportion of over-60-year-olds of the world’s population will nearly double from 12% to 22% [1]. China is one of the fastest aging countries with the largest number of elderly. By the end of 2015, there were 144 million people aged 65 years or older living in China, accounting for one-fifth of the world’s elderly population [2]. According to the U.S. Census Bureau, it will take China only 26 years to double the percentage of the elderly in their population, from 7% in 2000 to 14% in 2026 [3]. Improving the health level of the elderly to attain healthy aging has become an important issue in order to relieve the intense pressure brought by the aging world [4,5].

With the development of medicine and the medical sciences, health is now defined as the physical, mental, and social well-being, not merely the absence of disease or infirmity. Accordingly, health-related quality of life (HRQOL) is a multidimensional construct describing individuals’ perceptions of their physical, psychological, and social functioning [6]. Studying the HRQOL and its related factors in the elderly has been recognized as an important issue for health management and policy decisions [7,8,9].

Lifestyle factors are the most basic and modifiable individual variables that are connected to the HRQOL. Many previous studies have studied the relationship between the HRQOL and each lifestyle factor individually: smoking status [10,11], drinking status [12], sleep problems [13], exercise level [14], leisure activities [15], and so on. However, there were a few studies that combined them together to explore the relative contributions of different lifestyle factors to the HRQOL in the elderly. In this study, five different aspects of lifestyle factors were combined together to explore their relative importance for the HRQOL of the elderly. The results from this study will provide helpful information regarding the choice of effective lifestyle intervention to promote the HRQOL of the elderly. Furthermore, this study involves urban, rural, and institutionalized participants, which can represent different older people to some extent.

## 2. Materials and Methods 

### 2.1. Subjects and Data Collection

This study included three different subgroups of the elderly: the urban, rural, and institutionalized elderly. Compared with the community-dwelling elderly, the institutionalized elderly lead a highly-structured collective lifestyle that is obviously different from those who live in their own home in a community [16], while for community residents, those from urban and rural areas also have different lifestyles. Living in rural areas has been linked with a lower education level, poor economic status, less developed transportation, and harmful health-related behaviors [17,18]. Thus, considering the different characteristics among them, samples were collected from these three populations. 

Community participants were recruited from three representative urban communities and 11 rural villages, and institutionalized participants were recruited from 11 nursing homes in Xi’an, China. All the local target residents at selected communities or villages were informed of the study, and only those who came to the designated locations and met the inclusion criteria were involved. In nursing homes, one was included only if he/she meet the inclusion criteria. The inclusion criteria were: (1) aged 65 years or older; (2) community participants who lived in selected areas >5 years or institutionalized participants who lived in the selected nursing home >1 month; and (3) willingness to participate. At least 500 (which was larger than the minimum sample size suggested by the WHOQOL-OLD project protocol [19]) eligible respondents were selected from urban/rural areas, respectively, but only 266 eligible respondents were selected from the 11 nursing homes.

The survey was conducted face-to-face from July to November, 2014. Researchers who participated in this survey were graduate students in our department, and they were all formally trained and passed a uniform examination before the survey began. All procedures performed in this study involving human participants were in accordance with the ethical standards of the Xi’an Jiaotong University Ethics Committee (no. 2014-38). Written informed consent was obtained from each participant prior to the start of the study.

### 2.2. Instruments

#### 2.2.1. Health-Related Quality of Life Measures

We applied the Chinese (mainland) SF-36v2 Health Survey and the simplified Chinese WHOQOL-OLD module in this study to measure the HRQOL of the elderly. 

The SF-36 is one of the most widely-used generic HRQOL measures in the world [20]. It contains 36 items, and except for the one single item relating to health transition (HT), the remaining 35 items could be grouped into the following eight health domains: physical functioning (PF), role-physical (RP), bodily pain (BP), general health (GH), vitality (VT), social functioning (SF), role-emotional (RE), and mental health (MH). The eight domains could be further aggregated into two summary measures: physical component summary (PCS) and mental component summary (MCS). The standard (four-week recall) Chinese (mainland) version, provided by Quality Metric Incorporated (Lincoln, RI, USA), was used in this study. The 2009 U.S. general population norms were used as references due to the lack of general mainland Chinese population norms, but the equivalence of the SF-36 summary scales in the Chinese population has been proved [21]. Thus, all domains and summaries were calculated as norm-based scores, which have the means of 50 and standard deviations (SD) of 10. For all domains and summaries, higher scores indicated better HRQOL.

The WHOQOL-OLD module is an elderly-specific HRQOL measure which contains 24 items, which are divided into the following six domains: sensory abilities (SAB); autonomy (AUT); past, present, and future activities (PPF); social participation (SOP); death and dying (DAD); and intimacy (INT). Each domain provides a summary score and an overall score is calculated from the set of 24 items. The transformed scores ranging from 0–100 were used, with higher scores indicating a better HRQOL. The simplified Chinese version was provided by the WHOQOL group research center in China. Its reliability and validity were tested and confirmed in the Chinese population [22]. 

#### 2.2.2. Socio-Demographic and Lifestyle Questionnaires

Several socio-demographic information categories were included, such as gender, age, education level, and personal monthly income. Lifestyle information included smoking and drinking status, diet status (determined by the fresh fruit consumption frequency, on-time meal frequency, and food control), sleep status (determined by on-time sleep frequency, sleep disorders prevalence, and taking sleeping pills), exercise and labor status (determined by the frequency of their physical exercise, housework, and/or physical labor participation), and leisure time activities. According to the original design, the 11 detailed leisure time activities were transformed into six groups. The first group was named “individual sedentary pastime”, which contained watching TV/listening to the radio and reading books/newspapers; the second group was named “individual outdoor activities”, which referred to outdoor walking/fishing; the third group was named “individual non-developmental interest”, which included hand-making for fun and planting flowers; the fourth group was named “developmental interest”, which included singing/dancing/instrument-playing and writing/handwriting/collection/appreciation; the fifth group was named “interactive entertainment”, which included talking with others, playing chess or cards, and playing with a pet; and the last group referred to travel. If one was often engaged in one of the 11 leisure time activities, he/she scored one point accordingly, otherwise he/she scored zero. By adding up the scores of the according leisure time activities, the six groups of leisure time activity scores were obtained. 

### 2.3. Statistical Analysis

The participants were considered as a whole group, and were also divided into three sub-groups: urban, rural, and institutionalized elderly. For categorical data, we used relative numbers (frequency and proportion) to describe them and used chi-square analysis to compare their differences. For age, we used the mean and SD to describe it and used a one-way ANOVA to compare the difference among the subgroups. Scores were compared among the three subgroups using a one-way ANOVA. If significant differences were found, pairwise multiple comparisons were conducted to further examine the differences between the subgroups. Hierarchical linear regression analyses were performed to identify the relative contributions of different lifestyle factors on the HRQOL in the elderly. Independent variables included all characteristics shown in Table 1. Among them, age, education level, personal monthly income, and the six groups of leisure time activity scores were included as continuous values. Other categorical variables were included in the form of dummy variables. Different aspects of lifestyle factors were added into the regression model step by step. In model 1, only the basic characteristics were included; in model 2, the smoking and drinking status were added; in model 3, three diet-related factors were added; in model 4, three sleep-related factors were added; in model 5, three exercise-and-labor-related factors were added; and in model 6, the six groups of leisure time activities were added. Data analyses were carried out in the SPSS 13.0 statistical software (SPSS Inc., Chicago, IL, USA), with a *p* value less than 0.05 being the statistically significant level. 

## 3. Results

### 3.1. Characteristics of Participants

The final study sample consisted of 1369 elderly participants, including 542 participants from urban areas, 561 from rural areas, and 266 from nursing homes. Table 1 shows the participant characteristics. Of the whole sample, 60% of respondents were female, the mean age was 73.32 years, 49.9% had only finished primary school education or had no schooling and 40.5% had a personal monthly income of less than 500 Chinese Yuan (CNY). There were statistical differences in characteristics among the three subgroups, except for gender. Institutionalized persons were older than the urban and rural participants. The rural participants’ education level and personal monthly income was much lower compared with those of the other two subgroups.

### 3.2. Distributions of Health-Related Behaviors and Lifestyle

The distributions of health-related behaviors and lifestyle of the participants are shown in Table 1. Most were non-smokers (70.3%), non-drinkers (76.5%), had on-time meals (95.5%), and controlled their food intake (83.4%), slept on time (88.1%), and seldom took sleeping pills (91.6%), did physical exercise (69.7%), did housework (65.4%), and seldom participated in physical labor (69.7%). On leisure time activities, most elderly participants engaged in an individual sedentary pastime (85.2%), outdoor activities (73.3%), interactive entertainment (79.6%); and did not engage in individual non-developmental interest (55.7%), developmental interest (83.0%), and travel (77.0%). Furthermore, 43.1% ate fresh fruit frequently and 22.2% frequently suffered from sleep disorders. 

There were many statistical differences among the subgroups. In rural participants, more were smokers and their fresh fruit consumption frequency was the lowest. They performed physical exercise less, but participated in physical labors more and took sleeping pills less. Institutionalized participants had the largest proportion of abstainers, ate and slept regularly, and did less housework. However, they had a higher elderly proportion who frequently suffered from sleep disorders and took sleeping pills frequently. The urban elderly participated more in diverse activities. Except for developmental interests, their participation rates on other leisure groups were the highest among the subgroups. A high proportion of the institutionalized elderly spent their leisure time on developmental interests, and they had the extremely low elderly proportion who engaged in individual non-developmental interest.

### 3.3. Health-Related Quality of Life Scores

Table 2 presents the SF-36v2 and WHOQOL-OLD scores of the total participants and by subgroups. The PCS score was lower than 50 and the MCS score was higher than 50, which indicated a relatively poor physical health and better mental health than the general population. Among the subgroups, institutionalized elderly scored the lowest on the PCS, relatively lower on the MCS. On the eight SF-36v2 health domains, there were no differences on the BP and GH domains, but the institutionalized subgroup scored lower than the urban and rural subgroups on the PF, VT, and SF domains. The urban subgroup scored higher than the other two counterparts on the RP, SF, RE, and MH domains. For the WHOQOL-OLD module, the total score of the overall sample was 73.01, with the highest score on the DAD (88.31) domain and lowest on the INT (63.42) and SOP (68.29) domains. The institutionalized elderly reported the lowest total score, and urban elderly reported the highest. The urban subgroup scored higher than the other two subgroups on the SAB, AUT, and INT domains. The rural participants scored higher than the other subgroups on the PPF and SOP domains. There was no difference among the three subgroups on the DAD domain.

### 3.4. Relative Contributions of Different Lifestyle Factors to the HRQOL

In Table 3, which determines the physical component of the elder participants’ quality of life (PCS), all included factors explained 29.9% of the variance. It can be seen that the R^2^ changed the most (0.116) when the three exercise-and-labor-related factors were added. The physical HRQOL was positively associated with a higher education level, being a drinker, and higher scores on developmental interest, interactive entertainment, and travel. It was negatively associated with being female, seldom eating fresh fruit, suffering from sleep disorders, taking sleeping pills, seldom participating in physical exercise, sometimes or seldom doing housework, and sometimes or seldom participating in physical labor. Among the three subgroups (data not shown in table), all the included factors explained 34% variance of the urban respondents’ physical HRQOL, with exercise-and-labor-related factors (0.109), and sleep-related factors (0.095) causing the largest R^2^ change. For the rural subgroup, 27.4% of the physical HRQOL variance was explained. Among the different factors, exercise and labor (0.110) caused the largest R^2^ change. For the institutionalized subgroup, 41.1% of the physical HRQOL variance was explained by all the included factors, with exercise and labor (0.164) causing the largest R^2^ change.

For the mental component (Table 4), the included factors explained 21.8% of the variance in all. Sleep (0.054), leisure-time-activity-related factors (0.053) caused the largest R^2^ change. When all the factors were added, the mental HRQOL was positively associated with older age, higher education level, and higher scores of four leisure time activity groups. It was negatively associated with seldom eating fresh fruit, seldom having meals on time, suffering from sleep disorders, taking sleeping pills sometimes, and doing physical exercise sometimes/seldom. The included factors explained 31.1%, 25.6%, and 23.4% of the variance for the urban, rural and institutionalized subgroups respectively. And there is heterogeneity on the contributions of different lifestyle factors to the mental HRQOL among the subgroups. For the urban subgroup, sleep caused the largest R^2^ change (0.102), then was diet (0.063), leisure time activities (0.061), and exercise and labor (0.058) that caused nearly the same amount of R^2^ change. For the rural subgroup, sleep-related factors also caused the largest R^2^ change (0.085). However, among the subsequent three-factor sets, exercise and labor were not included, as it only caused an R^2^ change of 0.015. For the institutionalized elderly, it was exercise-and-labor-related factors that caused the largest R^2^ change (0.068). Next was diet (0.044) and leisure-time-activity-related factors (0.041), Sleep-related factors only caused an R^2^ change of 0.033 when they were added into the regression. 

For elderly-specific HRQOL, measured by the WHOQOL-OLD, the factors explained 32.2% of the variance in total (Table 5). Among the different factors, when leisure-time-activity-related factors were added, the R^2^ changed the most (0.119); next came exercise-and-labor-related factors (0.078). When all the factors were added, the elderly-specific HRQOL was positively associated with a higher education level, higher scores of five leisure time activity groups, and negatively associated with seldom eating fresh fruit, seldom having meals on time, suffering from sleep disorders, and sometimes or seldom doing physical exercise or housework. The included factors explained 37.8%, 29.5%, and 43.9% of the variance for the urban, rural, and institutionalized subgroups, respectively. Among the different factors, the leisure-time-activity-related factors caused the largest R^2^ change for the three subgroups of older people (urban: 0.146; rural: 0.084; institutionalized: 0.148). The next relatively important factor sets were not the same among the three subgroups. For urban people, it was sleep-related factors (0.089) and exercise-and-labor-related factors (0.070); for rural people, it was exercise-and-labor-related factors (0.079) and diet-related factors (0.058); and for the institutionalized elderly it was exercise-and-labor-related factors that caused the second highest R^2^ change (0.146), then were diet (0.060) and sleep (0.055) related factors.

## 4. Discussion

In general, based on the results of the present study, we might conclude that the HRQOL of participants was rather poor. Compared with older people from other parts of China, Shanghai’s elderly reported a higher PCS score of around 50, and a comparative MCS score of around 54 [23], while a large urban sample of the elderly from the Liaoning province reported even higher levels with average PCS and MCS scores of 53.7 ± 21.5 (mean ± SD) and 58.9 ± 18.9, respectively [24]. However, compared to the U.S. and Canadian norms of the elderly [25,26], the HRQOL level was similar to them. A large sample study (4316 respondents) on the quality of life in older adults was carried out by the WHOQOL Group and involved 20 countries from around the world. They reported higher overall WHOQOL-OLD scores than the elderly in our study [27]. 

One reason to explain these findings could be that the low income of most participants does not adequately cover their living expenses and, thus, they would face multiple problems that influence the quality of their life [28]. Another reason might be that a number of institutionalized older people who had obviously poor physical health were included [29,30] and certainly reported poor HRQOL compared with the other two subgroups. These results strongly suggest that administrators should anticipate greater healthcare demands from institutionalized populations. To further compare the HRQOL between urban and rural subgroups, the physical and elderly-specific HRQOL were at a comparable level, but the mental HRQOL of the elderly in rural areas was significantly lower. This may be explained by the lower education level of rural Chinese people and the low income, which may force them to work hard to farm their land in order to secure their basic livelihood [31,32].

Among the included lifestyle factors, except for smoking, and sleeping on time, the others were related to the HRQOL of the elderly to varying degrees. This result emphasized the importance of including interventions of lifestyle modification in healthy aging policy [33]. As for the different lifestyle factors, the hierarchical regression models showed the relative importance of them on the HRQOL of the elderly. Exercise-and-labor-related factors were the most important contributors to physical HRQOL, while sleep-related and leisure-time-activity-related factors contributed most to the mental HRQOL. Furthermore, leisure-time-activity-related factors contributed most to the elderly-specific HRQOL. Thus, to be most effective with improvements in physical health and physical HRQOL, interventions should focus on the exercise and labor aspect. As much of the previous literature has concluded, involvement in regular exercise elicited a number of favorable responses, ranging from the prevention of a number of functional declines to providing psychological benefits related to preserving cognitive function, and alleviating depressive symptoms and behaviors [14,34,35]. In order to improve the mental and elderly-specific HRQOL, both of which emphasize subjective feeling, people should focus on the leisure time activity aspect because most of the elderly do not work anymore. Thus, leisure time activities became an important part of elderly life and provide them with life value and meaning [36]. Through participating in leisure time activities, people can generate positive emotions, acquire additional skills and knowledge, and they may also build connections with other people if they participate in interactive entertainment [37]. Apart from leisure time activities, sleep-related factors also play an important role in predicting the mental HRQOL of the elderly, and this was in accord with earlier research [13]. 

Among the different subgroups, the relative importance of different lifestyle factors was also different. The exercise and labor aspect should be especially emphasized in the institutionalized subgroup. Furthermore, leisure time activities explained a large part of the variance of the HRQOL measured by the WHOQOL-OLD. In the urban subgroup, apart from leisure time activities and exercise and labor, sleep was the most critical aspects that influence the HRQOL. In the rural subgroup, sleep and diet were relatively important predictors of the HRQOL, apart from exercise and labor and leisure time activities. Thus, when carrying out interventions of lifestyle modification toward different people, implementers should concentrate on different aspects. 

Apart from lifestyle factors, other significant personal characteristics associated with the HRQOL were gender (only on MCS), age (only on PCS), and education level. The signs of significant characteristics are mostly in line with the literature. [25,38,39,40,41]

There were three major limitations of this research. Firstly, our study was implemented only in one area and the sample was not randomized, which limited the generalizability of the results for the whole of mainland China. Secondly, it would be ideal to compare the SF-36 results with the norm of the Chinese general population; however, the norm is unavailable. Thirdly, the factors included were limited and we were not able to examine environmental and social factors that may contribute to the HRQOL at the same time.

## 5. Conclusions

The findings of this study add to the growing body of evidence that lifestyle factors play a significant role in the HRQOL which, in turn, indicates the importance of including interventions of lifestyle modification in healthy aging policies. Additionally, the results showed that different lifestyle factors contributed to elderly HRQOL differently. Thus, to be most effective at the improvement of physical health and physical HRQOL, interventions should focus on the exercise and labor aspect. On the other hand, to improve the mental and elderly-specific HRQOL, both of which emphasize subjective feeling, related interventions should focus on the leisure time activity aspect. There was heterogeneity among the urban, rural, and institutionalized older people. Thus, the implementation of lifestyle interventions should concentrate on different aspects when dealing with different populations.

## Figures and Tables

**Table 1:** Characteristics of participants and distributions of lifestyle factors ^#^.

	Overall (*n* = 1369)	Urban (*n* = 542)	Rural (*n* = 561)	Institutionalized (*n* = 266)	*p*
Gender *n* (%)					0.1803
Male	547(40.0)	206(38.0)	222(39.6)	119(44.7)	
Female	822(60.0)	336(62.0)	339(60.4)	147(55.3)	
Age (years)					<0.0001
Mean ± SD	73.32 ± 6.32	73.88 ± 6.23	70.27 ± 4.99	78.59 ± 6.80	
Education level *n* (%)					<0.0001
No schooling	238(17.4)	79(14.6)	113(20.1)	46(17.4)	
Primary school	444(32.5)	95(17.5)	282(50.3)	67(25.3)	
Middle school	297(21.7)	113(20.8)	131(23.4)	53(20.0)	
High school	217(15.9)	138(25.5)	31(5.5)	48(18.1)	
College or higher	172(12.6)	117(21.6)	4(0.7)	51(19.2)	
Personal monthly income *n* (CNY, %)					<0.0001
<500	552(40.5)	60(11.1)	459(81.8)	33(12.6)	
500–2999	433(31.7)	230(42.4)	85(15.2)	118(45.2)	
≥3000	379(27.8)	252(46.5)	17(3.0)	110(42.1)	
Smoking status *n* (%)					<0.0001
Non-smokers	963(70.3)	394(72.7)	386(68.8)	183(68.8)	
Ex-smokers	162(11.8)	83(15.3)	29(5.2)	50(18.8)	
Current smokers	244(17.8)	65(12.0)	146(26.0)	33(12.4)	
Drinking status *n* (%)					0.0004
Nondrinkers	1047(76.5)	412(76.0)	430(76.6)	205(77.1)	
Abstainers	129(9.4)	38(7.0)	52(9.3)	39(14.7)	
Drinkers	193(14.1)	92(17.0)	79(14.1)	22(8.3)	
Fresh fruit consumption frequency *n* (%)					<0.0001
Frequently	590(43.1)	339(62.5)	136(24.2)	115(43.2)	
Sometimes	420(30.7)	102(18.8)	243(43.3)	75(28.2)	
Seldom	359(26.2)	101(18.6)	182(32.4)	76(28.6)	
Have meals on time *n* (%)					0.0007
Usually	1308(95.5)	517(95.4)	525(93.6)	266(100.0)	
Sometimes	44(3.2)	16(3.0)	28(5.0)	0(0.0)	
Seldom	17(1.2)	9(1.7)	8(1.4)	0(0.0)	
Food control *n* (%)					0.2330
Usually	1142(83.4)	448(82.7)	469(83.6)	225(84.6)	
Sometimes	63(4.6)	19(3.5)	32(5.7)	12(4.5)	
Seldom	164(12.0)	75(13.8)	60(10.7)	29(10.9)	
Sleep on time *n* (%)					0.0006
Usually	1206(88.1)	473(87.3)	481(85.7)	252(94.7)	
Sometimes	101(7.4)	40(7.4)	56(10.0)	5(1.9)	
Seldom	62(4.5)	29(5.4)	24(4.3)	9(3.4)	
Suffer from sleep disorders *n* (%)					0.0507
Frequently	304(22.2)	112(20.7)	116(20.7)	76(28.6)	
Sometimes	351(25.6)	140(25.8)	155(27.6)	56(21.1)	
Seldom	714(52.2)	290(53.5)	290(51.7)	134(50.4)	
Take sleeping pills *n* (%)					0.0007
Frequently	40(2.9)	18(3.3)	8(1.4)	14(5.3)	
Sometimes	75(5.5)	35(6.5)	19(3.4)	21(7.9)	
Seldom	1254(91.6)	489(90.2)	534(95.2)	231(86.8)	
Do physical exercise *n* (%)					<0.0001
Regularly	954(69.7)	410(75.6)	348(62.0)	196(73.7)	
Sometimes	133(9.7)	32(5.9)	78(13.9)	23(8.6)	
Seldom	282(20.6)	100(18.5)	135(24.1)	47(17.7)	
Do housework *n* (%)					<0.0001
Usually	895(65.4)	402(74.2)	448(79.9)	45(16.9)	
Sometimes	115(8.4)	48(8.9)	48(8.6)	19(7.1)	
Seldom	359(26.2)	92(17.0)	65(11.6)	202(75.9)	
Physical labor participation *n* (%)					<0.0001
Usually	273(19.9)	13(2.4)	256(45.6)	4(1.5)	
Sometimes	142(10.4)	23(4.2)	110(19.6)	9(3.4)	
Seldom	954(69.7)	506(93.4)	195(34.8)	253(95.1)	
Individual sedentary pastime score *n* (%)					<0.0001
0	203(14.8)	26(4.8)	137(24.4)	40(15.0)	
1	740(54.1)	238(43.9)	357(63.6)	145(54.5)	
2	426(31.1)	278(51.3)	67(11.9)	81(30.5)	
Individual outdoor activities score *n* (%)					<0.0001
0	366(26.7)	99(18.3)	188(33.5)	79(29.7)	
1	1003(73.3)	443(81.7)	373(66.5)	187(70.3)	
Individual non-developmental interest score *n* (%)					<0.0001
0	763(55.7)	213(39.3)	331(59.0)	219(82.3)	
1	479(35.0)	254(46.9)	187(33.3)	38(14.3)	
2	127(9.3)	75(13.8)	43(7.7)	9(3.4)	
Developmental interest score *n* (%)					<0.0001
0	1136(83.0)	434(80.1)	507(90.4)	195(73.3)	
1	207(15.1)	92(17.0)	51(9.1)	64(24.1)	
2	26(1.9)	16(3.0)	3(0.5)	7(2.6)	
Interactive entertainment score *n* (%)					0.0061
0	279(20.4)	116(21.4)	94(16.8)	69(25.9)	
1	749(54.7)	291(53.7)	312(55.6)	146(54.9)	
2	314(22.9)	124(22.9)	139(24.8)	51(19.2)	
3	27(2.0)	11(2.0)	16(2.9)	69(25.9)	
Travel *n* (%)					<0.0001
0	1054(77.0)	325(60.0)	494(88.1)	235(88.3)	
1	315(23.0)	217(40.0)	67(11.9)	31(11.7)	

^#^ One missing on education level and five missing on personal monthly income (in the institutionalized subgroup).

**Table 2:** Scores the SF-36v2 survey and WHOQOL-OLD module.

	Overall (*n* = 1369)	Urban (*n* = 542)	Rural (*n* = 561)	Institutionalized (*n* = 266)	*p*
SF-36v2					
Physical functioning (PF)	48.06 ± 9.93	48.92 ± 8.54	49.97 ± 8.91	42.31 ± 12.25	<0.0001
Role-physical (RP)	48.41 ± 10.86	51.40 ± 9.04	46.61 ± 11.61	46.12 ± 11.25	<0.0001
Bodily pain (BP)	50.66 ± 12.17	51.40 ± 11.71	50.38 ± 12.52	49.75 ± 12.31	0.1490
General health (GH)	45.76 ± 11.86	45.47 ± 12.03	45.85 ± 11.70	46.13 ± 11.85	0.7354
Vitality (VT)	56.37 ± 11.10	56.82 ± 10.51	56.83 ± 11.24	54.46 ± 11.76	<0.0077
Social functioning (SF)	50.95 ± 10.27	53.10 ± 8.88	50.12 ± 10.49	48.31 ± 11.53	<0.0001
Role-emotional (RE)	50.23 ± 9.61	52.70 ± 7.42	48.33 ± 10.56	49.22 ± 10.37	<0.0001
Mental health (MH)	53.88 ± 10.33	55.14 ± 9.41	52.88 ± 11.29	53.43 ± 9.77	<0.0010
Physical component summary (PCS)	47.05 ± 9.95	47.87 ± 9.27	47.76 ± 9.79	43.87 ± 11.00	<0.0001
Mental component summary (MCS)	54.38 ± 9.92	56.12 ± 8.40	52.77 ± 10.90	54.25 ± 10.10	<0.0001
WHOQOL-OLD					
Sensory abilities (SAB)	74.74 ± 22.30	77.72 ± 20.55	72.17 ± 22.99	74.06 ± 23.58	0.0002
Autonomy (AUT)	71.23 ± 19.93	73.99 ± 20.32	70.10 ± 18.55	68.00 ± 21.27	0.0001
Past, present and future activities (PPF)	72.10 ± 14.94	71.83 ± 14.99	74.52 ± 14.10	67.53 ± 15.47	<0.0001
Social participation (SOP)	68.29 ± 16.45	66.90 ± 16.20	70.58 ± 15.51	66.31 ± 18.26	0.0001
Death and dying (DAD)	88.31 ± 18.32	87.00 ± 18.85	89.45 ± 18.36	88.56 ± 16.99	0.0831
Intimacy (INT)	63.42 ± 23.99	68.23 ± 22.01	60.90 ± 25.24	58.93 ± 23.56	<0.0001
WHOQOL-OLD total	73.01 ± 11.99	74.28 ± 12.08	72.95 ± 11.46	70.56 ± 12.54	0.0002

**Table 3:** Hierarchical linear regression models on the physical component summary score of the SF-36v2.

	Model 1	Model 2	Model 3	Model 4	Model 5	Model 6
Gender (female vs. male)	−3.220 **	–3.426 **	−3.713 **	−2.899 **	−3.242 **	−3.345 **
Age	−0.243 **	−0.224 **	−0.213 **	−0.209 **	−0.037	−0.012
Education level	0.171	0.153	0.365	0.462	0.442	0.641 *
Personal monthly income	0.466	0.397	−0.074	0.242	0.899 *	0.538
Smoking status (vs. non-smokers)						
Ex-smokers		−2.113 *	−1.988 *	−1.554	−0.912	−1.138
Current smokers		−0.998	−0.700	−0.624	−0.330	−0.482
Drinking status (vs. nondrinkers)						
Abstainers		−1.024	−0.701	−1.056	−1.267	−1.204
Drinkers		3.922 **	3.869 **	3.574 **	2.579 **	2.033 **
Fresh fruit consumption frequency (vs. frequently)						
Sometimes			−1.794 **	−1.696 **	−1.482 *	−1.008
Seldom			−3.905 **	−3.551 **	−2.364 **	−1.856 **
Have meals on time (vs. usually)						
Sometimes			0.296	1.350	−0.112	0.559
Seldom			−2.418	−1.006	−1.145	−0.049
Food control (vs. usually)						
Sometimes			−0.902	−0.835	−0.963	−0.525
Seldom			1.518	1.507	1.035	1.334
Sleep on time (vs. usually)						
Sometimes				0.068	0.310	−0.004
Seldom				−1.413	−0.859	−0.683
Suffer from sleep disorders (vs. seldom)						
Sometimes				−2.291 **	−2.309 **	−2.151 **
Frequently				−4.429 **	−3.632 **	−3.254 **
Take sleeping pills (vs. seldom)						
Sometimes				−3.965 **	−2.985 **	−2.821 **
Frequently				−4.331 **	−3.792 *	−3.794 **
Do physical exercise (vs. regularly)						
Sometimes					−0.823	−0.025
Seldom					−5.117 **	−4.021 **
Do housework (vs. usually)						
Sometimes					−2.326 **	−2.218 *
Seldom					−5.604 **	−5.134 **
Physical labor participation (vs. usually)						
Sometimes					−2.655 **	−2.311 *
Seldom					−3.703 **	−3.835 **
Individual sedentary pastime						0.534
Individual outdoor activities						0.975
Individual non-developmental interest						−0.219
Developmental interest						1.716 **
Interactive entertainment						0.961 **
Travel						2.743 **
Constant	69.609 **	68.615 **	71.093 **	70.953 **	62.753 **	58.376 **
F value	16.036 **	12.311 **	9.993 **	11.999 **	18.846 **	17.713 **
R^2^ (adjusted)	0.045 (0.042)	0.068 (0.062)	0.094 (0.085)	0.152 (0.139)	0.268 (0.254)	0.299 (0.282)
R^2^ change	0.045	0.023	0.026	0.058	0.116	0.031

** *p* < 0.01; * *p* < 0.05.

**Table 4:** Hierarchical linear regression models on the mental component summary score of the SF-36v2.

	Model 1	Model 2	Model 3	Model 4	Model 5	Model 6
Gender (female vs. male)	−0.654	−0.481	−0.619	0.179	−0.347	−0.510
Age	0.193 **	0.198 **	0.182 **	0.182 **	0.183 **	0.196 **
Education level	0.624 *	0.615 *	0.819 **	0.893 **	0.880 **	1.082 **
Personal monthly income	1.455 **	1.388 **	0.945 *	1.124*	0.731	0.398
Smoking status (vs. non-smokers)						
Ex-smokers		0.137	0.429	0.785	0.852	0.517
Current smokers		−0.195	0.300	0.288	0.574	0.086
Drinking status (vs. nondrinkers)						
Abstainers		−0.574	−0.209	−0.347	−0.402	−0.057
Drinkers		1.513	1.528	1.368	1.207	0.625
Fresh fruit consumption frequency (vs. frequently)						
Sometimes			−2.597 **	−2.456 **	−1.895 **	−1.214
Seldom			−3.233 **	−2.875 **	−2.304 **	−1.567 *
Have meals on time (vs. usually)						
Sometimes			−3.916 **	−2.771	−2.173	−0.980
Seldom			−6.739 **	−5.127 *	−5.876 *	−4.523 *
Food control (vs. usually)						
Sometimes			−3.756 **	−3.650 **	−2.904*	−1.878
Seldom			−0.072	−0.141	−0.143	0.268
Sleep on time (vs. usually)						
Sometimes				−0.029	0.292	−0.183
Seldom				−1.134	−0.998	−0.940
Suffer from sleep disorders (vs. seldom)						
Sometimes				−2.352 **	−2.283 **	−1.982 **
Frequently				−5.161 **	−4.980 **	−4.417 **
Take sleeping pills (vs. seldom)						
Sometimes				−3.459 **	−3.305 **	−3.110 **
Frequently				1.036	1.060	0.981
Do physical exercise (vs. regularly)						
Sometimes					−3.784 **	−2.576 **
Seldom					−3.152 **	−1.384*
Do housework (vs. usually)						
Sometimes					−1.250	−0.854
Seldom					−1.569 *	−0.642
Physical labor participation (vs. usually)						
Sometimes					−0.446	−0.006
Seldom					1.583 *	1.332
Individual sedentary pastime						1.252 **
Individual outdoor activities						2.362 **
Individual non-developmental interest						0.154
Developmental interest						0.444
Interactive entertainment						1.847 **
Travel						2.293 **
Constant	40.257 **	39.586 **	44.251 **	44.592 **	45.991 **	39.466 **
F value	13.740 **	7.416 **	8.399 **	10.371 **	10.170 **	11.603 **
R^2^ (adjusted)	0.039 (0.036)	0.042 (0.036)	0.080 (0.071)	0.134 (0.121)	0.165 (0.149)	0.218 (0.199)
R^2^ change	0.039	0.003	0.038	0.054	0.031	0.053

** *p* < 0.01; * *p* < 0.05.

**Table 5:** Hierarchical linear regression models of the total score of the WHOQOL-OLD.

	Model 1	Model 2	Model 3	Model 4	Model 5	Model 6
Gender (female vs. male)	0.073	0.776	0.445	1.466	0.846	0.437
Age	−0.103	−0.085	−0.097	−0.090	0.012	0.046
Education level	0.096	0.050	0.375	0.504	0.479	1.032 **
Personal monthly income	0.623	0.525	−0.153	0.188	0.316	−0.465
Smoking status (vs. non-smokers)						
Ex-smokers		−0.556	−0.181	0.328	0.807	0.052
Current smokers		0.110	0.768	0.812	1.265	0.333
Drinking status (vs. nondrinkers)						
Abstainers		0.282	0.795	0.501	0.237	0.613
Drinkers		3.343 **	3.346 **	3.050 **	2.199 *	1.203
Fresh fruit consumption frequency (vs. frequently)						
Sometimes			−3.362 **	−3.223 **	−2.567 **	−1.425 *
Seldom			−5.462 **	−4.963 **	−3.677 **	−2.324 **
Have meals on time (vs. usually)						
Sometimes			−4.453 *	−3.166	−3.321	−1.152
Seldom			−9.486 **	−7.792 **	−8.424 **	−5.498 *
Food control (vs. usually)						
Sometimes			−3.426 *	−3.477 *	−2.896	−1.466
Seldom			1.362	1.297	0.920	1.776 *
Sleep on time (vs. usually)						
Sometimes				0.392	1.029	0.170
Seldom				−0.708	−0.123	−0.090
Suffer from sleep disorders (vs. seldom)						
Sometimes				−2.636 **	−2.526 **	−2.109 **
Frequently				−6.499 **	−5.760 **	−4.753 **
Take sleeping pills (vs. seldom)						
Sometimes				−4.240 **	−3.590 **	−3.413 **
Frequently				−3.032	−2.798	−3.224
Do physical exercise (vs. regularly)						
Sometimes					−4.924 **	−2.748 **
Seldom					−6.218 **	−3.353 **
Do housework (vs. usually)						
Sometimes					−4.706 **	−3.937 **
Seldom					−4.206 **	−2.521 **
Physical labor participation (vs. usually)						
Sometimes					−1.722	−1.085
Seldom					−1.336	−1.550
Individual sedentary pastime						2.942 **
Individual outdoor activities						1.979 **
Individual non-developmental interest						0.804
Developmental interest						2.808 **
Interactive entertainment						3.756 **
Travel						2.872 **
Constant	79.513 **	76.719 **	82.757 **	82.534 **	79.282 **	67.828 **
F value	0.978	1.970 *	6.372 **	9.576 **	13.081 **	19.738 **
R^2^ (adjusted)	0.003 (0.000)	0.012 (0.006)	0.062 (0.052)	0.125 (0.112)	0.203 (0.187)	0.322 (0.306)
R^2^ change	0.003	0.009	0.051	0.063	0.078	0.119

** *p* < 0.01; * *p* < 0.05.

## References

[1] Ageing and Health. http://www.who.int/mediacentre/factsheets/fs404/en/.

[2] Statistical Bulletin for the Development of Social Services in 2015. http://www.mca.gov.cn/article/zwgk/mzyw/201607/20160700001136.shtml.

[3] Kinsella K., Victoria A.V. (2001). An Aging World: 2001.

[4] Active Ageing: A Policy Framework. www.who.int/hpr/ageing/ActiveAgeingPolicyFrame.pdf.

[5] China Publishes a White Paper on Its Undertakings for the Aged. http://www.china.org.cn/english/China/191990.htm.

[6] Schipper H., Clinch J.J., Olweny C.L.M., Spilker B. (1996). Quality of life studies: Definitions and conceptual issues. Quality of Life and Pharmacoeconomics in Clinical Trial.

[7] Anderson K.L., Burckhardt C.S. (1999). Conceptualization and measurement of quality of life as an outcome variable for health care intervention and research. J. Adv. Nurs..

[8] Higginson I.J., Carr A.J. (2001). Measuring quality of life: Using quality of life measures in the clinical setting. BMJ.

[9] Lauper P., Versteegen U. (1992). The importance of quality of life in policy decisions: New endpoints in medical outcome assessment. Pharm. Med..

[10] Wilson D., Parsons J., Wakefield M. (1999). The Health-Related Quality-of-Life of Never Smokers, Ex-smokers, and Light, Moderate, and Heavy Smokers. Prev. Med..

[11] Tillmann M., Silcock J. (1997). A comparison of smokers’ and ex-smokers’ health-related quality of life. J. Public Health Med..

[12] Byles J., Young A., Furuya H., Parkinson L. (2006). A Drink to Healthy Aging: The Association between Older Women’s Use of Alcohol and Their Health-Related Quality of Life. J. Am. Geriatr. Soc..

[13] Schubert C.R., Cruickshanks K.J., Dalton D.S., Klein B.E.K., Klein R., Nondahl D.M. (2002). Prevalence of sleep problems and quality of life in an older population. Sleep.

[14] Balboa-Castillo T., León-Muñoz L.M., Graciani A., Rodríguez-Artalejo F., Guallar-Castillón P. (2011). Longitudinal association of physical activity and sedentary behavior during leisure time with health-related quality of life in community-dwelling older adults. Heal. Qual. Life Outcomes.

[15] Silverstein M., Parker M.G. (2002). Leisure Activities and Quality of Life among the Oldest Old in Sweden. Res. Aging.

[16] Fiveash B. (1998). The experience of nursing home life. Int. J. Nurs. Pract..

[17] Sicular T., Ximing Y., Gustafsson B., Shi L. (2007). The Urban-Rural Income Gap and Inequality in China.

[18] Magilvy J.K., Congdon J.G. (2000). The Crisis Nature of Health Care Transitions for Rural Older Adults. Public Health Nurs..

[19] Power M., Quinn K., Schmidt S. (2005). Development of the WHOQOL-Old module. Qual. Life Res..

[20] Haywood K.L., Garratt A.M., Fitzpatrick R. (2006). Quality of life in older people: A structured review of self-assessed health instruments. Expert Rev. Pharm. Outcomes Res..

[21] Lam C.L.K., Tse E.Y.Y., Gandek B., Fong D.Y.T. (2005). The SF-36 summary scales were valid, reliable, and equivalent in a Chinese population. J. Clin. Epidemiol..

[22] Liu R., Wu S., Hao Y., Gu J., Fang J., Cai N., Zhang J. (2013). The Chinese version of the world health organization quality of life instrument-older adults module (WHOQOL-OLD): Psychometric evaluation. Health Qual. Life Outcomes.

[23] Wang R., Wu C., Zhao Y., Yan X., Ma X., Wu M., Liu W., Gu Z., Zhao J., He J. (2008). Health related quality of life measured by SF-36: A population-based study in Shanghai, China. BMC Public Health.

[24] Sun W., Aodeng S., Tanimoto Y., Watanabe M., Han J., Wang B., Yu L., Kono K. (2015). Quality of life (QOL) of the community-dwelling elderly and associated factors: A population-based study in urban areas of China. Arch. Gerontol. Geriatr..

[25] Maruish M.E. (2011). User’s Manual for the SF-36v2 Health Survey.

[26] Hopman W.M., Towheed T., Anastassiades T., Tenenhouse A., Poliquin S., Berger C., Joseph L., Brown J.P., Murray T.M., Adachi J.D. (2000). Canadian normative data for the SF-36 health survey. CMAJ.

[27] Chachamovich E., Fleck M., Laidlaw K., Power M. (2008). Impact of Major Depression and Subsyndromal Symptoms on Quality of Life and Attitudes Toward Aging in an International Sample of Older Adults. Gerontologist.

[28] Deaton A. (2008). Income, Health, and Well-Being around the World: Evidence from the Gallup World Poll. J. Econ. Perspect..

[29] Gu D., Dupre M.E., Liu G. (2007). Characteristics of the institutionalized and community-residing oldest-old in China. Soc. Sci. Med..

[30] Rockwood K., Howlett S.E., MacKnight C., Beattie B.L., Bergman H., Hébert R., Hogan D.B., Wolfson C., McDowell I. (2004). Prevalence, Attributes, and Outcomes of Fitness and Frailty in Community-Dwelling Older Adults: Report From the Canadian Study of Health and Aging. J. Gerontol. A Biol. Sci. Med. Sci. Ser. A.

[31] Araya R. (2003). Education and income: Which is more important for mental health?. J. Epidemiol. Community Health.

[32] Zhou B., Chen K., Wang J., Wang H., Zhang S., Zheng W. (2010). Quality of Life and Related Factors in the Older Rural and Urban Chinese Populations in Zhejiang Province. J. Appl. Gerontol..

[33] Peel N.M., McClure R.J., Bartlett H.P. (2005). Behavioral determinants of healthy aging. Am. J. Prev. Med..

[34] Mazzeo R.S., Cavanagh P., Evans W.J., Fiatarone M., Hagberg J., McAuley E., Startzell J. (1998). Exercise and Physical Activity for Older Adults. Med. Sci. Sports Exerc..

[35] Bize R., Johnson J.A., Plotnikoff R.C. (2007). Physical activity level and health-related quality of life in the general adult population: A systematic review. Prev. Med..

[36] McKinnon A.L. (1992). Time use for Self Care, Productivity, and Leisure among Elderly Canadians. Can. J. Occup. Ther..

[37] Brajša-Žganec A., Merkaš M., Šverko I. (2011). Quality of Life and Leisure Activities: How do Leisure Activities Contribute to Subjective Well-Being?. Soc. Indic. Res..

[38] Nilsson J., Rana A.K., Kabir Z.N. (2006). Social capital and quality of life in old age: Results from a cross-sectional study in rural Bangladesh. J. Aging Health.

[39] Paskulin L., Vianna L., Molzahn A.E. (2009). Factors associated with quality of life of Brazilian older adults. Int. Nurs. Rev..

[40] De Belvis A., Avolio M., Spagnolo A., Damiani G., Sicuro L., Cicchetti A., Ricciardi W., Rosano A. (2008). Factors associated with health-related quality of life: The role of social relationships among the elderly in an Italian region. Public Health.

[41] Yahaya N., Abdullah S.S., Momtaz Y.A., Hamid T.A. (2010). Quality of Life of Older Malaysians Living Alone. Educ. Gerontol..

